# PTree: pattern-based, stochastic search for maximum parsimony phylogenies

**DOI:** 10.7717/peerj.89

**Published:** 2013-06-25

**Authors:** Ivan Gregor, Lars Steinbrück, Alice C. McHardy

**Affiliations:** 1 Max-Planck Research Group for Computational Genomics and Epidemiology, Max-Planck Institute for Informatics, Saarbrücken, Germany; 2 Department of Algorithmic Bioinformatics, Heinrich-Heine-University Düsseldorf, Düsseldorf, Germany

**Keywords:** Phylogeny reconstruction, Maximum parsimony, Local search, Stochastic search

## Abstract

Phylogenetic reconstruction is vital to analyzing the evolutionary relationship of genes within and across populations of different species. Nowadays, with next generation sequencing technologies producing sets comprising thousands of sequences, robust identification of the tree topology, which is optimal according to standard criteria such as maximum parsimony, maximum likelihood or posterior probability, with phylogenetic inference methods is a computationally very demanding task. Here, we describe a stochastic search method for a maximum parsimony tree, implemented in a software package we named PTree. Our method is based on a new pattern-based technique that enables us to infer intermediate sequences efficiently where the incorporation of these sequences in the current tree topology yields a phylogenetic tree with a lower cost. Evaluation across multiple datasets showed that our method is comparable to the algorithms implemented in PAUP* or TNT, which are widely used by the bioinformatics community, in terms of topological accuracy and runtime. We show that our method can process large-scale datasets of 1,000–8,000 sequences. We believe that our novel pattern-based method enriches the current set of tools and methods for phylogenetic tree inference. The software is available under: http://algbio.cs.uni-duesseldorf.de/webapps/wa-download/.

## Introduction

Phylogenetic analysis infers the evolutionary relationships among genes from within or across distinct populations of different species. As input, we are often given a set of aligned genetic sequences for which we wish to work out the pattern of ancestry. This analysis plays an important role in, for example, drug or vaccine development (McHardy & Adams, 2009; Russell et al., 2008). With the cost of sequencing decreasing rapidly due to next generation sequencing technologies (Metzker, 2010), more and more sequences are becoming available and deposited in sequence repositories such as GenBank (Benson et al., 2011). Therefore, new, fast and accurate methods capable of handling large-scale datasets are required (Sanderson, 2007).

Commonly used methods for phylogenetic inference fall into four categories: distance-based methods, maximum parsimony, maximum likelihood and Bayesian methods. Distance-based methods are data clustering methods that consider only the pairwise measure of evolutionary distances among sequences. Maximum parsimony assumes that the correct phylogenetic tree is the one requiring the smallest number of evolutionary events to explain the input sequences. A maximum likelihood method requires a substitution model to assess the probability of particular phylogenetic trees, where it aims to find a tree with the highest likelihood with respect to the given substitution model. Bayesian methods rely on probabilistic models of sequence evolution, like maximum likelihood tree inference. Different from maximum likelihood estimates, however, trees are constructed for instance based on the consensus of a set of trees sampled from the highest probability regions of the posterior distribution over evolutionary model parameters and trees. There are several software packages that implement variants of these methods: PHYLIP (Felsenstein, 2005), PAUP* (Swofford, 2002), TNT (Goloboff, Farris & Nixon, 2008), PSODA (Carroll et al., 2009), POY (Varón, Vinh & Wheeler, 2010), MEGA5 (Tamura et al., 2011) and MRBAYES (Huelsenbeck & Ronquist, 2001).

Maximum likelihood and Bayesian methods are considered to be the most accurate, and are nowadays relatively fast due to the computational capacity of modern computers and methodological advances (Guindon & Gascuel, 2003; Huelsenbeck & Ronquist, 2001; Stamatakis, Ludwig & Meier, 2005; Zwickl, 2006). However, these methods depend on large numbers of parameters that have to be estimated, such as branch lengths, tree topology, parameters of the substitution model and site-specific rate variations, which make them even more computationally demanding. Although maximum likelihood methods are widely used, Kück et al. (2012) showed that the high confidence in maximum likelihood trees is not always justified for certain tree shapes. Distance-based methods, such as the unweighted pair group method with arithmetic mean (UPGMA) and the neighbor-joining (NJ) algorithm, are quite fast, with runtime depending only quadratically and cubically, respectively, on the number of input sequences (Saitou & Nei, 1987; Sokal & Michener, 1958). However, distance-based methods are less accurate than all other techniques. We here have focused on the maximum parsimony criterion for tree inference, since this approach has a reasonable trade-off between speed and accuracy and is still considered as an important optimality criterion for the evaluation of phylogenetic trees (Steel & Penny, 2000), especially for datasets at lower evolutionary divergence (Guindon & Gascuel, 2003). Moreover, Goloboff, Catalano & Farris (2009) showed that maximum parsimony can be successfully employed in the analysis of a large-scale dataset of 73,060 taxa. Additionally, they found that long branch attraction (Felsenstein, 1978), which can cause problems when using maximum parsimony, does not play an important role in determining the general structure of the tree when the taxonomic space is sufficiently covered by the sample. Furthermore, a tree computed by a maximum parsimony method can be used as a good starting solution for a subsequent run of a maximum likelihood method (Sundberg et al., 2008).

Finding the optimal solution to the maximum parsimony problem is NP-hard (Graham, 1982). The research on exact maximum parsimony searches has resulted in two methods (Kasap & Benkrid, 2011; White & Holland, 2011). Although these methods make use of parallel hardware, they are applicable only on small datasets (<40 taxa). A conjecture as to whether it would be possible to build exact maximum parsimony trees from several trees on fewer taxa turned out to be invalid in general. Fischer (2012) showed that maximum parsimony trees are not hereditary, i.e., an instance of the maximum parsimony problem cannot, in general, be reduced to the same problem on fewer taxa. Therefore, approximation algorithms have been designed to identify good, near optimal solutions. In a commonly used approach, tree rearrangement operators such as nearest neighbor interchange (NNI), subtree pruning and regrafting (SPR), or tree bisection and reconnection (TBR) are applied to define a state space of tree topologies, which is then traversed in local searches with strategies such as best first, best first with backtracking, simulated annealing or genetic algorithms (Felsenstein, 2003). Operators introducing more extensive tree rearrangements such as TBR and SPR usually yield a better solution than less extensive tree rearrangement operators such as NNI; however, this comes with the cost of longer runtimes. Some work has also been devoted to exploring how to modify the size of a neighborhood of a tree that is being explored during one step of a local search algorithm to ensure that the algorithm converges to a near optimal solution and avoids local minima. Goëffon, Richer & Hao (2008) showed that the progressive neighborhood, where the size of the neighborhood is shrinking towards the end of a local search algorithm, results in faster convergence to a near optimal solution.

Genetic algorithms, methods that mimic the process of natural evolution, can be also employed for the maximum parsimony problem (Hill et al., 2005). Genetic algorithms can be also combined with local search methods such that each tree resulting from a recombination operation is subsequently subjected to a local search method that tries to improve its cost. Such algorithms are called memetic algorithms. One implementation of a memetic algorithm is described in Richer, Goëffon & Hao (2009).

Greedy algorithms, which build a solution step by step, can be also employed to reconstruct a phylogenetic tree; such algorithms add one sequence at a time. However, the performance of these algorithms strongly depends on the order in which individual sequences are added. They are thus often combined with other strategies (e.g., local searches). One such an implementation is described in Ribeiro & Vianna (2005).

Here, we describe PTree, a pattern-based, stochastic search method for the maximum parsimony problem that can be applied for large datasets (∼8,000 taxa). The method is based on our pattern-based tree reconstruction method which is a new technique that can be used to reconstruct phylogenetic trees from genetic sequences; it also suggests how to explore the neighborhood of a tree in the local search. To investigate the performance of the method, we have compared it with the local search techniques implemented in the two widely used software packages PAUP* and TNT.

## Design and Methods

### Method overview

In this section, we will describe our method in a bottom-up fashion. First, we will describe the key idea of our method, which is the *Repeated Substitution Pattern* (RSP). Given a local tree topology, i.e., a particular internal node of a tree, its parent and children, the *RSP* defines how the sequences of potential new intermediate nodes, which improve the parsimony cost of the respective local tree topology, can be inferred. As a lot of intermediate nodes may be inferred for an internal node of a tree, we define how this number can be restricted in the subsection *Intermediates Sampling*. In particular, we aim to choose, primarily, intermediate nodes with the highest potential to lower the parsimony cost of a particular local tree topology. Then, we describe the *Pattern-Based Tree Reconstruction* method that reconstructs a phylogenetic tree topology from a sequence alignment, employing the *RSP* and *Intermediates Sampling*. In our software *PTree* the *Pattern-Based Tree Reconstruction* method is employed as an operation in a local search algorithm to identify the most parsimonious tree topology. The *Randomization* of several steps in PTree for further performance improvement is described in the last subsection. In the following, we will use terms *node* and *sequence* interchangeably, as in our method, each node of a tree represents one DNA sequence.

### Repeated substitution pattern

**Figure 1:**
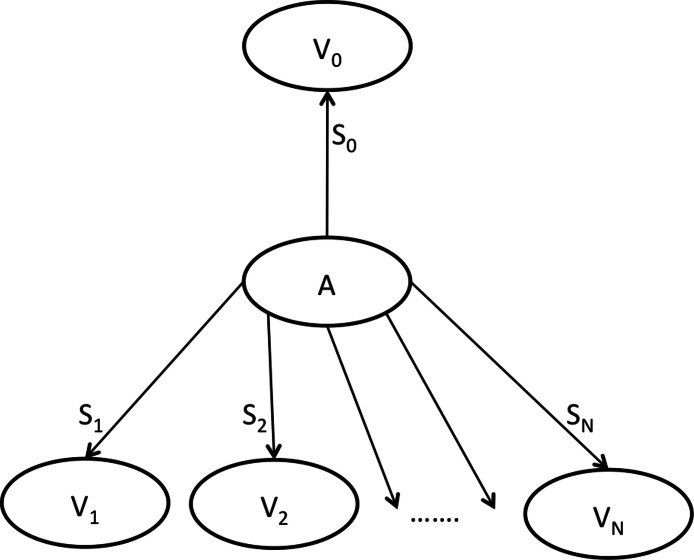
Local tree topology. *A* is an internal node, *V*_0_ its parent, *V*_1_…*V*_*N*_ are its children, and *S*_*i*_ represents the corresponding substitution sets.

The RSP defines how new intermediate sequences are inferred at an internal node of the tree, considering only a particular local tree topology. Let us consider the local tree topology depicted in Fig. 1, where *A* is an internal node, *V*_0_ its parent and *V*_1_…*V*_*N*_ are its children, and let *S*_*i*_ be the substitution set of node *V*_*i*_ consisting of the differences between sequences of *V*_*i*_ and *A* (as seen from *A* to *V*_*i*_). Note that the arrow that goes from node *A* to its parent *V*_0_ does not represent the direction in which the local tree topology is rooted but it serves only for the purpose of the definition of the RSP. A repeated substitution set is defined as the non-empty intersection (*Y*_*k*_ = *S*_*i*_∩*S*_*j*_) between two distinct substitution sets *S*_*i*_, *S*_*j*_ for *i*, *j*∈[0…*N*] and *i* ≠ *j*. Based on this, we infer a new intermediate sequence *I*_*k*_ for all distinct non-empty repeated substitution sets *Y*_*k*_ at internal node *A*. A new intermediate node *I*_*k*_ originates from the sequence of *A* and subsequent application of the substitutions contained in the corresponding repeated substitution set *Y*_*k*_. We say that a RSP was found at internal node *A*, if there is at least one non-empty repeated substitution set *Y*_*k*_ (i.e., the intersection between at least two distinct substitution sets is non-empty). If the RSP was found at a particular internal node, the output of the application of the RSP to the internal node of the tree is a non-empty list that contains all new intermediate nodes that can be inferred at this internal node. In the following, we will denote the application of the RSP to internal node *A* as a call of function *rPattern(A)* that returns a list of new intermediate nodes (*I*_*k*_).

**Figure 2:**
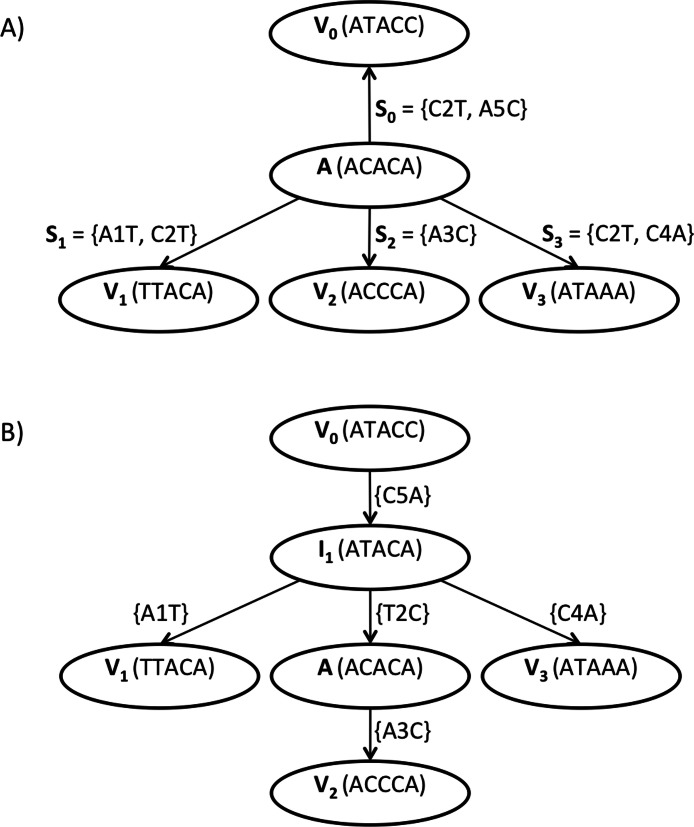
Repeated substitution pattern example. (A) depicts a local tree topology where node *A* is an internal node that represents DNA sequence (*A* *C* *A* *C* *A*), *V*_0_ its parent, *V*_1_…*V*_3_ its children, and *S*_0_…*S*_3_ are corresponding substitution sets. The repeated substitution pattern is found at node *A* since the intersection of at least two substitution sets is non-empty, namely: *S*_0_∩*S*_1_ = *S*_0_∩*S*_3_ = *S*_1_∩*S*3 = {*C*2*T*} = *Y*_1_. Thus, new candidate intermediate node *I*_1_ (*A* *T* *A* *C* *A*) originates from *A* (*A* *C* *A* *C* *A*) by applying mutations in *Y*_1_ = {*C*2*T*}. Note that the arrow that goes from node *A* to its parent *V*_0_ does not represent the direction in which the local tree topology is rooted but it serves only for the purpose of the definition of the repeated substitution pattern. (B) depicts an expected local tree topology after intermediate node *I*_1_ is added to the tree topology. After *I*_1_ is added, the cost of the local tree topology (i.e., the number of substitutions) decreases from 7 to 5.

Figure 2A depicts an example of a local tree topology where node *A* is an internal node that represents DNA sequence *(ACACA)*, *V*_0_ its parent that represents DNA sequence *(ATACC)*. *V*_1_…*V*_3_ are children of *A* that represent DNA sequences *(TTACA)*, *(ACCCA)* and *(ATAAA)* respectively. Substitution sets are depicted at respective arrows as seen from internal node *A*. For instance, the differences in the sequences of *A* and *V*_0_ are described via substitution set *S*_0_. Sequences of *A* and *V*_0_ differ in two positions, namely, *C* is substituted by *T* at the second position and *A* is substituted by *C* at the fifth position. According to the definition of the RSP, we can see that the intersection of at least two substitution sets (with distinct indices) is non-empty, namely *S*_0_∩*S*_1_ = *S*_0_∩*S*_3_ = *S*_1_∩*S*_3_ = {*C*2*T*} = *Y*_1_. Thus, we can create a sequence of intermediate node *I*_1_, such that we apply the mutations in the intersection set *Y*_1_ = {*C*2*T*} to the DNA sequence of node *A(ACACA),* which results in intermediate sequence *I*_1_*(ATACA),* i.e., *rPattern(A)* returns a list of length one that contains *I*_1_. Figure 2B depicts an expected local tree topology after node *I*_1_ is added. After the new internal node *I*_1_ is added to the local tree topology, its cost decreases from 7 to 5. Thus, the application of the RSP enables the inference of new intermediate nodes that can refine the overall tree topology.

### Intermediates sampling

As the application of the RSP, i.e., application of the function *rPattern(A)* to an internal node *A* of degree *n* may produce up to *n*(*n*−1)/2 intermediate nodes, we restrict the number of intermediate nodes that can be inferred for a particular internal node with a two-step procedure.

**Step 1.** We define the maximum number of intermediate nodes that can be inferred at some internal node as a function of the degree of an internal node: *F*(*n*) = max(*round*(*c*_1_ *n*), *c*_2_) where *c*_1_ and *c*_2_ are fixed coefficients (e.g., *c*_1_ = 4.0 and *c*_2_ = 1). The purpose of this function is to allow the inference of more intermediate nodes at internal nodes of higher degree. Let *I*_*A*_ be the set of intermediate nodes that can be inferred at internal node *A* (i.e., *I*_*A*_≔*rPattern*(*A*)) and *M*≔*F* *(degree of A)*. To restrict |*I*_*A*_| (i.e., the number of intermediate nodes that will be inferred at *A*), we choose at most *M* intermediate nodes out of *I*_*A*_ with the biggest cost decrease, i.e., we compute for each intermediate node from *I*_*A*_ the cost decrease if a node was included in the local tree topology regardless of the other candidate intermediate sequences. For instance, the cost decrease after node *I*_1_ is included in the local tree topology in Fig. 2 is two. Note that we perform the choice of intermediate nodes at random if we have more options for how to choose intermediate nodes with the biggest cost decrease. Given *I*_*A*_, let us denote the set of at most *M* intermediate nodes with the biggest cost decrease selected in this step as *I*_*A*_*s*1_.

**Step 2.** Given *I*_*A*_*s*1_, the intermediate nodes selected in the first step, the corresponding internal node *A*, and its parent and children, we apply the following procedure:

(1) Compute the distance matrix (DM) based on pairwise evolutionary distances among the sequences.
(2) Compute the minimum spanning tree (MST) based on the DM.
(3) Remove intermediate nodes of degree one or two.
(4) Repeat steps (1) to (4) until no intermediate nodes can be removed in step (3).
(5) Return the list of intermediate nodes that remain in the MST; let us denote this list as *I*_*A*_*s*2_.

In the following, we will denote the application of intermediates sampling as a call of function *iSample* with a list of intermediate nodes and a respective internal node as its arguments. Thus, *iSample(rPattern(A), A)* is a list of restricted size *I*_*A*_*s*2_ that contains intermediate nodes with the highest potential to lower the parsimony cost of the local tree topology defined at internal node *A*.

### Pattern-based tree reconstruction

This method reconstructs a tree topology from a given sequence alignment that can represent both original input sequences that represent, for example, species, and the corresponding inferred intermediate ancestral sequences, where the latter are auxiliary and optional. Let *seqSet* be the set of the aligned input sequences. The method comprises seven steps:

(1) Initialize the distance matrix (DM), i.e., compute a neighbor-joining (NJ) tree based on pairwise evolutionary distances among the given input sequences *seqSet*. Set the DM to the path metric representing the distances in the inferred NJ tree, such that for each pair of the input sequences (i.e., leaf nodes of the NJ tree), the DM contains an entry that specifies their distance in the NJ tree. Here, we initialize the DM for the consequent minimum spanning tree (MST) computation in this way, since we observed that our method yields better results than if we computed the MST only based on the pairwise number of differences among the input sequences.
(2) Compute an MST from the DM. The MST represents a tree with the lowest cost considering only the given input sequences *seqSet*. The MST is an approximation of a minimum Steiner tree (Hwang, Richards & Winter, 1992); therefore it is a good starting point for the intermediate inference, in which we further improve the tree topology (i.e., lower its overall parsimony cost) by adding new intermediate nodes.
(3) Infer new candidate intermediate sequences based on the RSPs. Note that a candidate intermediate sequence denotes a sequence that can be added to the tree topology, while an intermediate sequence refers to a sequence that has been added to the current tree topology. All candidate intermediate sequences, inferred at this step, are collected by the depth-first search (DFS) algorithm, which efficiently traverses the whole tree and visits all internal nodes to identify RSPs. Thus, we get the list of all candidate intermediate sequences *cSeq* by calling function *iSample(rPattern(A), A*) for each internal node *A* of the tree and by concatenating the resulting lists. More precisely, new candidate intermediate sequences are inferred under consideration of a particular local tree topology, consisting of an arbitrary internal node, its parent, and its children. New candidate intermediate sequences are inferred if a RSP can be found for this local tree topology. Intermediates sampling, the two-step approach, is then employed to get only a restricted number of candidate intermediate nodes at a particular internal node. After all new candidate intermediate sequences have been collected, we remove duplicate candidate intermediate sequences from *cSeq* (duplicates to each other, or to sequences represented by nodes in the current tree, i.e., in *seqSet*). Note that inferred intermediate sequences are subsequently added to the sequence set for the tree inference, and can be placed as either internal or leaf nodes in the subsequent tree inference steps. Therefore, the inference of new intermediate sequences plays an important role.
(4) Merge the new candidate intermediate sequences *cSeq*, inferred in the previous step, with all sequences from the current tree *seqSet*. Let *seqSet* be the merged set. Iteratively recompute the distance matrix (DM), i.e., set the entries of the DM to specify the number of different characters between each pair of sequences from *seqSet*, recompute the MST according to the DM and remove intermediate nodes of degree one (i.e., leaf nodes) or two (superfluous internal nodes), as they are uninformative, until no (candidate) intermediate sequence can be removed. Note that the corresponding sequences are also removed from *seqSet*. The candidate intermediate sequences that remain in the current tree after this step become intermediate sequences and *seqSet* contains all sequences of the nodes of the current tree.
(5) Repeat steps (2) to (5) until no new candidate intermediate sequences can be found in step (3).
(6) For each internal node that represents an original input sequence (e.g., existing species), create a copy of the node and place it as a child node of the respective internal node that represents the same sequence, so that all original input sequences are represented by leaf nodes of the resulting tree.
(7) Return the resulting phylogenetic tree topology.

Let us denote the application of this pattern-based tree reconstruction method to the set of input sequences s*eqSet* as a call of function *pbTree(seqSet)* that returns a tree topology.

### PTree

Our software PTree employs the pattern-based tree reconstruction method as an operation in a local search for the most parsimonious tree topology. PTree searches for an optimal solution to the maximum parsimony problem by traversing the tree space of feasible solutions and keeping only the last feasible solution with the lowest parsimony cost that has been found so far.

In addition to the set of aligned input genetic sequences *seqSet*, PTree requires specification of the parameters *d*, *b* and *k*: The parameter *d* denotes the percentages of internal nodes to be deleted from the tree. To allow refinement of the tree topology, the search for the most parsimonious tree topology cannot terminate before *b* iterations overall (the burn-in phase) and before *k* iterations where no more parsimonious tree relative to the best tree found so far has been observed. For our experiments, we set *d* to 10%, *b* to 100 and *k* to 10.

PTree operates in three steps:

(1) The algorithm initializes the search with a neighbor-joining (NJ) tree inferred from the input sequence alignment *seqSet*. We set the *currentTree* to be this NJ tree. In PTree, we have incorporated a relaxed neighbor-joining algorithm with computational complexity O(N^2^logN) (Sheneman, Evans & Foster, 2006). Distance corrections based on the Jukes–Cantor (Jukes & Cantor, 1969) or the Kimura two-parameter model (Kimura, 1980) that are available in this implementation can optionally be performed, however, in our experiments we found no notable differences in results with different evolutionary models.
(2) Updating the current tree topology, i.e., delete *d*% of randomly chosen internal nodes from the *currentTree* and return *rSeq*, the set of sequences associated with remaining nodes (internal and leaf nodes). The set *rSeq* is passed to our pattern-based tree reconstruction method, i.e., the function *pbTree(rSeq)* returns a *newTree*. If the *newTree* has a lower parsimony cost than the *currentTree*, the *newTree* becomes the *currentTree*; else, the *currentTree* remains the same as it was.
(3) Repeat steps (2) and (3) until the number of iterations is larger than *b* and no update of the *currentTree* has been performed for the last *k* iterations. Then, return the *currentTree* as the final phylogenetic tree.

### Randomization

Randomization of some steps of our method substantially improves its accuracy. The MST algorithm, the NJ algorithm and the intermediates sampling are randomized. Since some edges of a graph can have the same weights and any of these edges can be added at a particular step of an MST algorithm, the MST is not unequivocally defined. Standard implementations of the MST algorithm usually return the same tree after two consecutive runs with the same input. Thus, we have modified the MST algorithm so that it is likely that two consecutive runs produce two different tree topologies with the same cost, using the same input sequences. At the point where the MST algorithm can add several distinct edges with the same cost (weight) to the current graph, it decides at random which edge will be added. There can be more options for choosing two clusters that will be merged at a particular step of an NJ algorithm, and thus the resulting NJ tree is also not unequivocally defined; therefore, we have employed a randomized version of the relaxed NJ algorithm (Sheneman, Evans & Foster, 2006). Different MST topologies facilitate inference of more various candidate intermediate sequences, which leads to better accuracy of the resulting phylogenetic trees, since we also explore the larger neighborhood of the current trees in the local search.

## Results

To validate the performance of PTree, we compared it with widely used methods on reference datasets of PhyML (Guindon & Gascuel, 2003) and RAxML (Stamatakis, Ludwig & Meier, 2005), as well as on alignments of concatenated Human Immunodeficiency Virus (HIV) reverse transcriptase and polymerase sequences from EuResist (2010). We tested for topological accuracy, cost optimality and runtime. All computations were done on a machine with 2.8 GHz Dual-Core AMD Opteron Processor 8820. The Java Virtual Machine was given 4GB of the main memory for the datasets up to 4,000 taxa and 8GB for the datasets of 8,000 taxa, and all programs were run in a single thread.

### Topological accuracy

**Figure 3:**
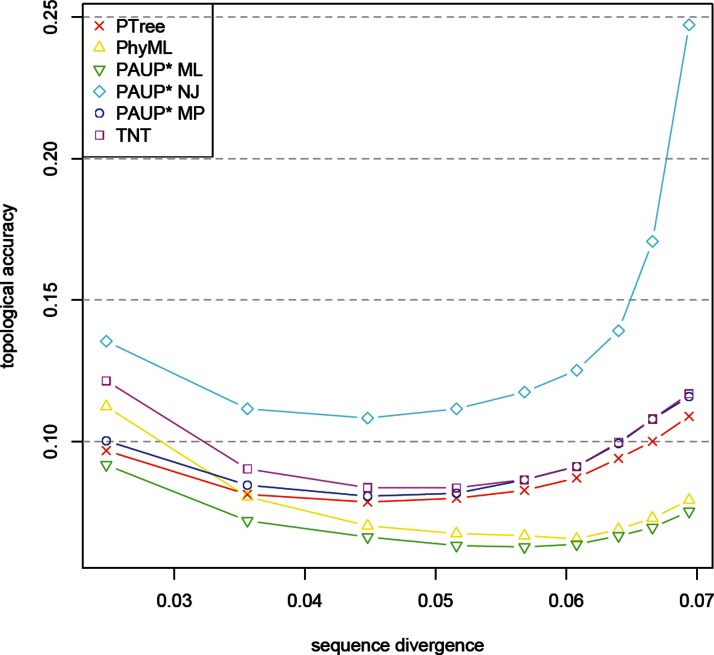
Topological accuracy. Comparison of topological accuracies of selected tree building methods as a function of sequence divergence using 5,000 datasets of 40 taxa with 500 bases each from Guindon & Gascuel (2003). PTree was run with 50 iterations and Jukes–Cantor correction enabled. PAUP* was run with the following settings: Neighbor Joining (NJ), maximum parsimony (MP; with the tree bisection and reconnection (TBR) branch swapping option used in the heuristic search), and maximum likelihood (ML; with the nearest neighbor interchange (NNI) branch swapping option used in the heuristic search). TNT was run with the subtree pruning and regrafting (SPR) branch swapping option used in the heuristic search.

Tests for topological accuracy were done in accordance to PhyML (Guindon & Gascuel, 2003) on 5,000 datasets with known topology where each dataset contained 40 taxa of 500bp length each. To compare the true and the reconstructed tree topologies, we used the Robinson-Foulds distance (Robinson & Foulds, 1981), although this measure is more conservative than other measures such as SPR or TBR, we chose it to allow a comparison with results shown for PhyML in Guindon & Gascuel (2003). Reference methods were PAUP* NJ, PAUP* maximum parsimony (with the TBR branch swapping option used in the heuristic search), PAUP* maximum likelihood (with the NNI branch swapping option used in the heuristic search), PhyML and TNT (with the SPR branch swapping option used in the heuristic search). The results are depicted in Fig. 3. As expected, the best results in terms of the Robinson–Foulds distance (Robinson & Foulds, 1981) between the inferred and true tree topology were achieved by the maximum likelihood methods, followed by the parsimony methods (including PTree). The worst results were yielded by NJ, the representative of a distance-based method. Furthermore, within the parsimony methods, PTree outperforms PAUP* used with the tree bisection and reconnection (TBR) heuristic, which is the most extensive and accurate search heuristic.

### Cost optimality and runtime

To assess the performance of PTree in terms of parsimony costs and runtime, we compared it with PAUP* NNI, SPR, TBR and TNT SPR heuristics on the reference datasets of RAxML, as well as on a dataset of HIV reverse transcriptase and polymerase sequences. For each dataset, we created seven subsets of different size (125–8,000 sequences). Sequences of the HIV datasets were ∼1,600bp in length and the sequences of the RAxML datasets were ∼1,200bp. For the RAxML subsets, we took the arb_10000 dataset, selected the first 8,000 sequences and then subsequently selected the first half of the remaining sequences for a new dataset. The HIV subsets were created in a similar fashion: we downloaded ∼8,000 reverse transcriptase and polymerase sequences from EuResist (2010), aligned them and repeatedly selected the first half of the remaining sequences as a new dataset. As two consecutive runs of PAUP* (NNI, SPR, or TBR) or PTree can result in different results, we identified the mean (average over multiple runs), minimum, and maximum parsimony costs and runtimes. Thus, PAUP* was run six times (except for the tests with the SPR and TBR heuristics with the RAxML dataset with 4,000 sequences that were run just once; the test with the TBR heuristic with the RAxML dataset with 2,000 sequences that was run twice; and the test with the TBR heuristic with the HIV dataset with 4,000 sequences that was run twice as well); PTree (run with 100 iterations and without distance correction) was run 10 times for all datasets. Some tests were done with lower number of runs due to the limited computational resources. The comparison of the resulting average parsimony costs is shown in Tables 1 and 3, and the comparison of the corresponding average runtimes is shown in Tables 2 and 4. The comparisons of the corresponding minimum and maximum parsimony costs, as well as the comparison of the minimum and maximum runtimes are shown in Tables S1–S8. Furthermore, to enable the comparison of different methods proportionally to the resulting parsimony costs and runtimes of PTree, Tables S9–S20 show the results of Tables 1–4 and Tables S1–S8, respectively, in terms of percentages. In terms of the average values, PTree outperforms PAUP* with the NNI heuristic search in terms of the parsimony costs, except for the 250-sequence HIV dataset, where PTree yields slightly higher costs. Moreover, PTree was also substantially faster than PAUP* NNI for large datasets with 1,000 or more sequences. Compared to PAUP* with the SPR or TBR heuristic and TNT with the SPR heuristic, PTree performs worse in all tests in terms of the parsimony costs. Nevertheless, both search heuristics implemented in PAUP* are usually unfeasible due to their high runtime on large datasets with more than 2,000 sequences. However, the SPR heuristic implemented in TNT is applicable for large datasets. While PTree was faster than TNT SPR with the HIV dataset with more than 4,000 sequences, PTree was slower than TNT SPR with the RAxML dataset. The best results in terms of runtime were gained by PAUP* NJ, which shows substantially decreased runtime compared to all tested methods. However, parsimony costs and topological accuracy suggest that NJ should be used only for some initial results.

**Table 1:** Average parsimony cost comparison using the HIV dataset. Comparison of average parsimony costs for the Neighbor Joining (NJ) algorithm, PTree, TNT with the subtree pruning and regrafting (SPR) search heuristic, and PAUP* with different search heuristics: nearest neighbor interchange (NNI), SPR, and tree bisection and reconnection (TBR), on different dataset sizes using concatenated sequences of HIV reverse transcriptase and polymerase with ∼1,600 bases each downloaded from EuResist (2010).

	Size of input dataset						
	125	250	500	1,000	2,000	4,000	8,000
**Method**							
NJ	6,298	11,789	21,911	42,383	79,567	152,546	289,472
PAUP* (NNI)	6,132	11,557	21,559	41,636	78,421	150,316	287,530
PTree	6,104	11,572	21,549	41,573	78,276	149,950	286,815
TNT (SPR)	6,078	11,487	21,401	41,250	77,556	148,527	283,921
PAUP* (SPR)	6,083	11,482	21,405	41,274	77,594	148,683	–
PAUP* (TBR)	6,080	11,476	21,381	41,235	77,537	148,601	–

**Table 2:** Average runtime comparison using the HIV dataset. Average runtime comparison for selected methods on different dataset sizes using concatenated sequences of HIV reverse transcriptase and polymerase with ∼1,600 bases each downloaded from EuResist (2010).

	Size of input dataset						
	125	250	500	1,000	2,000	4,000	8,000
**Method**							
NJ	0.2 s	0.2 s	0.5 s	2 s	9 s	35 s	9 m 13 s
PAUP* (NNI)	2.4 s	11.8 s	1 m 54 s	23 m 44 s	1 h 46 m	11 h 26 m	92 h 52 m
PTree	19.4 s	58.5 s	3 m 30 s	14 m 13 s	1 h 4 m	4 h 47 m	23 h 48 m
TNT (SPR)	4 s	14 s	1 m 24 s	6 m 38 s	41 m 2 s	6 h 34 m	33 h 38 m
PAUP* (SPR)	27.7 s	1 m 30 s	19 m 35 s	2 h 44 m	22 h	155 h 17 m	–
PAUP* (TBR)	27.7 s	4 m 33 s	28 m 43 s	5 h 2 m	35 h 49 m	190 h 31 m	–

**Table 3:** Average parsimony cost comparison using the RAxML dataset. Comparison of average parsimony costs for selected methods on different-sized datasets with sequence lengths of ∼1,200 bases from Stamatakis, Ludwig & Meier (2005).

	Size of input dataset						
	125	250	500	1,000	2,000	4,000	8,000
**Method**							
NJ	957	2,675	6,045	12,704	21,698	71,883	151,678
PAUP* (NNI)	946	2,650	5,970	12,489	21,188	70,391	148,718
PTree	934	2,607	5,898	12,299	20,951	70,247	148,423
TNT (SPR)	934	2,592	5,850	12,214	20,743	69,545	146,906
PAUP* (SPR)	934	2,597	5,852	12,203	20,718	69,508	–
PAUP* (TBR)	933	2,589	5,836	12,159	20,673	69,380	–

**Table 4:** Average runtime comparison using the RAxML dataset. Average runtime comparison for selected methods on different-sized datasets with sequence lengths of ∼1,200 bases from Stamatakis, Ludwig & Meier (2005).

	Size of input dataset						
	125	250	500	1,000	2,000	4,000	8,000
**Method**							
NJ	0.1 s	0.1 s	0.1 s	1 s	8 s	1 m	8 m 28 s
PAUP* (NNI)	1.4 s	11.4 s	1 m 30 s	17 m 7 s	2 h 51 m	28 h 33 m	139 h 49 m
PTree	7.5 s	38.5 s	2 m 14 s	6 m 43 s	23 m 56 s	2 h 10 m	11 h 10 m
TNT (SPR)	0.7 s	3 s	14 s	51 s	5 m 23 s	57 m 57 s	4 h 48 m
PAUP* (SPR)	17.7 s	2 m 44 s	19 m 51 s	2 h 5 m	60 h 23 m	>1 month	–
PAUP* (TBR)	41.2 s	4 m 56 s	39 m 22 s	4 h 38 m	252 h 42 m	>1 month	–

## Conclusion

PTree is a novel stochastic pattern-based search method for maximum parsimony trees. We compared our method with maximum parsimony tree inference using different local search methods with PAUP* and TNT, which are widely used by the bioinformatics community. Our tests showed that PTree outperformed NNI searches in terms of the topological accuracy and cost optimality of identified trees. PTree is also substantially faster than PAUP* NNI searches and comparable to TNT SPR for large datasets (1,000–8,000 sequences). The implementation of our current method is in Java, which is a platform independent programming language (only the NJ algorithm (Sheneman, Evans & Foster, 2006) was added as a C/C++ library). To further improve runtimes, we may reimplement some parts of the algorithm in C and add it as a C/C++ library using the Java Native Interface in the future. We found that more elaborate search heuristics, PAUP* SPR, PAUP* TBR and TNT SPR, yield trees with better parsimony costs than PTree, however they are often more computationally demanding.

Our pattern based tree reconstruction method could be also combined with other algorithms that compute phylogenetic trees. For instance, it could be employed in a memetic algorithm in several ways: as an operation in a local search algorithm, as a mutation operator or as a recombination operator. To recombine two trees, the pattern-based tree reconstruction method can be given all previously inferred internal nodes of both trees and all original input sequences as an input, which results in a new tree. Thus, our new pattern-based method enriches the set of current phylogeny reconstruction tools and methods.

## Supplemental Information

10.7717/peerj.89/supp-1 Table S1 Minimum parsimony cost comparison using the HIV dataset

10.7717/peerj.89/supp-2 Table S2 Average runtime comparison using the HIV dataset proportional to PTree

10.7717/peerj.89/supp-3 Table S3 Average parsimony cost comparison using the RAxML dataset proportional to PTree

10.7717/peerj.89/supp-4 Table S4 Average runtime comparison using the RAxML dataset proportional to PTree

10.7717/peerj.89/supp-5 Table S5 Minimum parsimony cost comparison using the HIV dataset proportional to PTree

10.7717/peerj.89/supp-6 Table S6 Minimum runtime comparison using the HIV dataset proportional to PTree

10.7717/peerj.89/supp-7 Table S7 Maximum parsimony cost comparison using the HIV dataset proportional to PTree

10.7717/peerj.89/supp-8 Table S8 Maximum runtime comparison using the HIV dataset proportional to PTree

10.7717/peerj.89/supp-9 Table S9 Minimum parsimony cost comparison using the RAxML dataset proportional to PTree

10.7717/peerj.89/supp-10 Table S10 Minimum runtime comparison using the RAxML dataset proportional to PTree

10.7717/peerj.89/supp-11 Table S11 Maximum parsimony cost comparison using the RAxML dataset proportional to PTree

10.7717/peerj.89/supp-12 Table S12 Minimum runtime comparison using the HIV dataset

10.7717/peerj.89/supp-13 Table S13 Maximum runtime comparison using the RAxML dataset proportional to PTree

10.7717/peerj.89/supp-14 Table S14 Maximum parsimony cost comparison using the HIV dataset

10.7717/peerj.89/supp-15 Table S15 Maximum runtime comparison using the HIV dataset

10.7717/peerj.89/supp-16 Table S16 Minimum parsimony cost comparison using the RAxML dataset

10.7717/peerj.89/supp-17 Table S17 Minimum runtime comparison using the RAxML dataset

10.7717/peerj.89/supp-18 Table S18 Maximum parsimony cost comparison using the RAxML dataset

10.7717/peerj.89/supp-19 Table S19 Maximum runtime comparison using the RAxML dataset

10.7717/peerj.89/supp-20 Table S20 Average parsimony cost comparison using the HIV dataset proportional to PTree
