# Supplementary material for: PTree: pattern-based, stochastic search for maximum parsimony phylogenies
Source: PeerJ. 2013 Jun 25;1:e89. doi: 10.7717/peerj.89 (PMC3698465; doi:10.7717/peerj.89)
Supplement: Table S1 [file peerj-01-89-s001.pdf]

		Size of input dataset						
		125	250	500	1,000	2,000	4,000	8,000
**Method**	NJ	6,298	11,789	21,911	42,383	79,567	152,546	289,472
	PAUP* (NNI)	6,099	11,537	21,534	41,578	78,317	150,208	287,336
	PTree	6,099	11,553	21,529	41,547	78,238	149,905	286,687
	TNT (SPR)	6,078	11,487	21,401	41,250	77,556	148,527	283,921
	PAUP* (SPR)	6,075	11,474	21,392	41,232	77,498	148,658	–
	PAUP* (TBR)	6,076	11,471	21,374	41,204	77,486	148,590	–
