# Supplementary material for: PTree: pattern-based, stochastic search for maximum parsimony phylogenies
Source: PeerJ. 2013 Jun 25;1:e89. doi: 10.7717/peerj.89 (PMC3698465; doi:10.7717/peerj.89)
Supplement: Table S2 [file peerj-01-89-s002.pdf]

		Size of input dataset						
		125	250	500	1,000	2,000	4,000	8,000
Method	NJ	1.031	0.342	0.238	0.234	0.234	0.203	0.645
	PAUP* (NNI)	12.371	20.171	54.286	166.940	165.625	239.024	390.196
	PTree	100	100	100	100	100	100	100
	TNT (SPR)	20.619	23.932	40.0	46.659	64.115	137.282	141.317
	PAUP* (SPR)	142.784	153.846	559.524	1,153.58	2,062.50	3,246.34	–
	PAUP* (TBR)	142.784	466.667	820.476	2,124.27	3,357.81	3,982.93	–
