# Supplementary material for: PTree: pattern-based, stochastic search for maximum parsimony phylogenies
Source: PeerJ. 2013 Jun 25;1:e89. doi: 10.7717/peerj.89 (PMC3698465; doi:10.7717/peerj.89)
Supplement: Table S3 [file peerj-01-89-s003.pdf]

		Size of input dataset						
		125	250	500	1,000	2,000	4,000	8,000
Method	NJ	102.463	102.608	102.492	103.293	103.565	102.329	102.193
	PAUP* (NNI)	101.285	101.649	101.221	101.545	101.131	100.205	100.199
	PTree	100	100	100	100	100	100	100
	TNT (SPR)	100	99.425	99.186	99.309	99.007	99.001	98.978
	PAUP* (SPR)	100	99.616	99.220	99.219	98.888	98.948	–
	PAUP* (TBR)	99.893	99.310	98.949	98.862	98.673	98.766	–
