# Supplementary material for: PTree: pattern-based, stochastic search for maximum parsimony phylogenies
Source: PeerJ. 2013 Jun 25;1:e89. doi: 10.7717/peerj.89 (PMC3698465; doi:10.7717/peerj.89)
Supplement: Table S4 [file peerj-01-89-s004.pdf]

		Size of an input dataset						
		125	250	500	1,000	2,000	4,000	8,000
**Method**	NJ	1.333	0.260	0.075	0.248	0.557	0.769	1.264
	PAUP* (NNI)	18.667	29.610	67.164	254.839	714.485	1,317.69	1,252.09
	PTree	100	100	100	100	100	100	100
	TNT (SPR)	9.333	7.792	10.448	12.655	22.493	44.577	42.985
	PAUP* (SPR)	236.000	425.974	888.806	1,861.04	15,137.9	>33,500.0	–
	PAUP* (TBR)	549.333	768.831	1,762.69	4,138.96	63,351.0	>33,500.0	–
