# Supplementary material for: PTree: pattern-based, stochastic search for maximum parsimony phylogenies
Source: PeerJ. 2013 Jun 25;1:e89. doi: 10.7717/peerj.89 (PMC3698465; doi:10.7717/peerj.89)
Supplement: Table S5 [file peerj-01-89-s005.pdf]

		Size of input dataset						
		125	250	500	1,000	2,000	4,000	8,000
**Method**	NJ	103.263	102.043	101.774	102.012	101.699	101.762	100.971
	PAUP* (NNI)	100	99.862	100.023	100.075	100.101	100.202	100.226
	PTree	100	100	100	100	100	100	100
	TNT (SPR)	99.656	99.429	99.405	99.285	99.128	99.081	99.035
	PAUP* (SPR)	99.606	99.316	99.364	99.242	99.054	99.168	–
	PAUP* (TBR)	99.623	99.290	99.280	99.174	99.039	99.123	–
