# Supplementary material for: PTree: pattern-based, stochastic search for maximum parsimony phylogenies
Source: PeerJ. 2013 Jun 25;1:e89. doi: 10.7717/peerj.89 (PMC3698465; doi:10.7717/peerj.89)
Supplement: Table S6 [file peerj-01-89-s006.pdf]

		Size of input dataset						
		125	250	500	1,000	2,000	4,000	8,000
**Method**	NJ	1.075	0.365	0.250	0.244	0.238	0.215	0.677
	PAUP* (NNI)	5.914	12.591	42.000	96.224	111.111	112.177	167.671
	PTree	100	100	100	100	100	100	100
	TNT (SPR)	21.505	25.547	42.000	48.477	65.132	145.387	148.273
	PAUP* (SPR)	50.538	125.912	249.000	635.810	1,207.94	1,025.46	–
	PAUP* (TBR)	81.183	273.723	479.000	1,235.08	2,868.25	4,080.44	–
