# Supplementary material for: PTree: pattern-based, stochastic search for maximum parsimony phylogenies
Source: PeerJ. 2013 Jun 25;1:e89. doi: 10.7717/peerj.89 (PMC3698465; doi:10.7717/peerj.89)
Supplement: Table S7 [file peerj-01-89-s007.pdf]

		Size of input dataset						
		125	250	500	1,000	2,000	4,000	8,000
**Method**	NJ	103.128	101.717	101.524	101.860	101.595	101.686	100.883
	PAUP* (NNI)	101.032	100.069	100.130	100.228	100.249	100.277	100.271
	PTree	100	100	100	100	100	100	100
	TNT (SPR)	99.525	99.111	99.161	99.137	99.027	99.007	98.949
	PAUP* (SPR)	99.722	99.146	99.226	99.332	99.197	99.143	–
	PAUP* (TBR)	99.689	99.077	99.147	99.176	99.077	99.064	–
