# Supplementary material for: PTree: pattern-based, stochastic search for maximum parsimony phylogenies
Source: PeerJ. 2013 Jun 25;1:e89. doi: 10.7717/peerj.89 (PMC3698465; doi:10.7717/peerj.89)
Supplement: Table S8 [file peerj-01-89-s008.pdf]

		Size of input dataset						
		125	250	500	1,000	2,000	4,000	8,000
Method	NJ	1.000	0.323	0.23	0.228	0.227	0.193	0.633
	PAUP* (NNI)	38.000	43.387	69.124	287.244	274.242	387.459	646.598
	PTree	100	100	100	100	100	100	100
	TNT (SPR)	20.000	22.581	38.710	45.330	62.172	130.033	138.694
	PAUP* (SPR)	520.000	206.452	737.327	1,763.10	3,454.55	5,425.74	–
	PAUP* (TBR)	194.000	793.548	1,163.59	2,924.83	5,342.42	3,895.71	–
