# Supplementary material for: PTree: pattern-based, stochastic search for maximum parsimony phylogenies
Source: PeerJ. 2013 Jun 25;1:e89. doi: 10.7717/peerj.89 (PMC3698465; doi:10.7717/peerj.89)
Supplement: Table S9 [file peerj-01-89-s009.pdf]

| | | Size of input dataset | | | | | | |
|---|---|---|---|---|---|---|---|---|
| | | 125 | 250 | 500 | 1,000 | 2,000 | 4,000 | 8,000 |
| Method | NJ | 102.572 | 102.766 | 102.719 | 103.647 | 103.679 | 102.393 | 102.232 |
| | PAUP* (NNI) | 100.429 | 101.114 | 100.612 | 101.558 | 100.889 | 100.162 | 100.069 |
| | PTree | 100 | 100 | 100 | 100 | 100 | 100 | 100 |
| | TNT (SPR) | 100.107 | 99.577 | 99.405 | 99.649 | 99.116 | 99.063 | 99.016 |
| | PAUP* (SPR) | 99.893 | 99.616 | 99.150 | 99.364 | 98.939 | 99.010 | – |
| | PAUP* (TBR) | 99.893 | 99.347 | 98.912 | 99.062 | 98.686 | 98.828 | – |
