# Supplementary material for: PTree: pattern-based, stochastic search for maximum parsimony phylogenies
Source: PeerJ. 2013 Jun 25;1:e89. doi: 10.7717/peerj.89 (PMC3698465; doi:10.7717/peerj.89)
Supplement: Table S10 [file peerj-01-89-s010.pdf]

		Size of an input dataset						
		125	250	500	1,000	2,000	4,000	8,000
**Method**	NJ	1.563	0.323	0.077	0.264	0.597	0.794	1.297
	PAUP* (NNI)	17.188	24.839	41.846	221.372	595.078	842.857	1,078.71
	PTree	100	100	100	100	100	100	100
	TNT (SPR)	10.937	9.677	10.769	13.456	24.087	45.992	44.104
	PAUP* (SPR)	225.000	354.839	703.077	1,139.84	8,612.98	>34,500.0	–
	PAUP* (TBR)	504.687	477.419	1,200.77	2,406.33	63,270.7	>34,500.0	–
