# Supplementary material for: PTree: pattern-based, stochastic search for maximum parsimony phylogenies
Source: PeerJ. 2013 Jun 25;1:e89. doi: 10.7717/peerj.89 (PMC3698465; doi:10.7717/peerj.89)
Supplement: Table S11 [file peerj-01-89-s011.pdf]

		Size of input dataset						
		125	250	500	1,000	2,000	4,000	8,000
**Method**	NJ	102.463	102.334	102.336	103.050	103.442	102.250	102.130
	PAUP* (NNI)	102.355	102.028	101.761	101.655	101.454	100.317	100.325
	PTree	100	100	100	100	100	100	100
	TNT (SPR)	100	99.158	99.035	99.075	98.889	98.925	98.917
	PAUP* (SPR)	100.535	99.541	99.374	99.173	98.856	98.872	–
	PAUP* (TBR)	100.321	99.120	99.035	98.824	98.651	98.690	–
