# Supplementary material for: PTree: pattern-based, stochastic search for maximum parsimony phylogenies
Source: PeerJ. 2013 Jun 25;1:e89. doi: 10.7717/peerj.89 (PMC3698465; doi:10.7717/peerj.89)
Supplement: Table S12 [file peerj-01-89-s012.pdf]

| | | Size of input dataset | | | | | | |
|---|---|---|---|---|---|---|---|---|
| | | 125 | 250 | 500 | 1,000 | 2,000 | 4,000 | 8,000 |
| **Method** | NJ | 0.2s | 0.2s | 0.5s | 2s | 9s | 35s | 9m 13s |
| | PAUP* (NNI) | 1.1s | 6.9s | 1m 24s | 13m 10s | 1h 10m | 5h 4m | 38h 2m |
| | PTree | 18.6s | 54.8s | 3m 20s | 13m 41s | 1h 3m | 4h 31m | 22h 41m |
| | TNT (SPR) | 4s | 14s | 1m 24s | 6m 38s | 41m 2s | 6h 34m | 33h 38m |
| | PAUP* (SPR) | 9.4s | 1m 9s | 8m 18s | 1h 27m | 12h 41m | 46h 19m | – |
| | PAUP* (TBR) | 15.1s | 2m 30s | 15m 58s | 2h 49m | 30h 7m | 184h 18m | – |
