# Supplementary material for: PTree: pattern-based, stochastic search for maximum parsimony phylogenies
Source: PeerJ. 2013 Jun 25;1:e89. doi: 10.7717/peerj.89 (PMC3698465; doi:10.7717/peerj.89)
Supplement: Table S13 [file peerj-01-89-s013.pdf]

		Size of an input dataset						
		125	250	500	1,000	2,000	4,000	8,000
**Method**	NJ	1.000	0.244	0.073	0.209	0.503	0.758	1.243
	PAUP* (NNI)	21.000	33.415	91.241	242.380	908.291	1,733.33	1,468.58
	PTree	100	100	100	100	100	100	100
	TNT (SPR)	7.000	7.317	10.219	10.647	20.289	43.902	42.291
	PAUP* (SPR)	213.000	543.902	1,385.40	1,954.07	17,638.2	>33,000.0	–
	PAUP* (TBR)	513.000	1,280.49	2,715.33	5,060.54	60,995.0	>33,000.0	–
