# Supplementary material for: PTree: pattern-based, stochastic search for maximum parsimony phylogenies
Source: PeerJ. 2013 Jun 25;1:e89. doi: 10.7717/peerj.89 (PMC3698465; doi:10.7717/peerj.89)
Supplement: Table S14 [file peerj-01-89-s014.pdf]

Method		Size of input dataset						
		125	250	500	1,000	2,000	4,000	8,000
Method	NJ	6,298	11,789	21,911	42,383	79,567	152,546	289,472
	PAUP* (NNI)	6,170	11,598	21,610	41,704	78,513	150,432	287,716
	PTree	6,107	11,590	21,582	41,609	78,318	150,016	286,938
	TNT (SPR)	6,078	11,487	21,401	41,250	77,556	148,527	283,921
	PAUP* (SPR)	6,090	11,491	21,415	41,331	77,689	148,731	–
	PAUP* (TBR)	6,088	11,483	21,398	41,266	77,595	148,612	–
