# Supplementary material for: PTree: pattern-based, stochastic search for maximum parsimony phylogenies
Source: PeerJ. 2013 Jun 25;1:e89. doi: 10.7717/peerj.89 (PMC3698465; doi:10.7717/peerj.89)
Supplement: Table S15 [file peerj-01-89-s015.pdf]

		Size of input dataset						
		125	250	500	1,000	2,000	4,000	8,000
**Method**	NJ	0.2s	0.2s	0.5s	2s	9s	35s	9m 13s
	PAUP* (NNI)	7.6s	26.9s	2m 30s	42m 2s	3h 1m	19h 34m	156h 48m
	PTree	20s	1m 2s	3m 37s	14m 38s	1h 6m	5h 3m	24h 15m
	TNT (SPR)	4s	14s	1m 24s	6m 38s	41m 2s	6h 34m	33h 38m
	PAUP* (SPR)	1m 44s	2m 8s	26m 40s	4h 18m	38h	274h	–
	PAUP* (TBR)	38.8s	8m 12s	42m 5s	7h 8m	58h 46m	196h 44m	–
