# Supplementary material for: PTree: pattern-based, stochastic search for maximum parsimony phylogenies
Source: PeerJ. 2013 Jun 25;1:e89. doi: 10.7717/peerj.89 (PMC3698465; doi:10.7717/peerj.89)
Supplement: Table S16 [file peerj-01-89-s016.pdf]

		Size of input dataset						
		125	250	500	1,000	2,000	4,000	8,000
**Method**	NJ	957	2,675	6,045	12,704	21,698	71,883	151,678
	PAUP* (NNI)	937	2,632	5,921	12,448	21,114	70,317	148,468
	PTree	933	2,603	5,885	12,257	20,928	70,203	148,366
	TNT (SPR)	934	2,592	5,850	12,214	20,743	69,545	146,906
	PAUP* (SPR)	932	2,593	5,835	12,179	20,706	69,508	–
	PAUP* (TBR)	932	2,586	5,821	12,142	20,653	69,380	–
