# Supplementary material for: PTree: pattern-based, stochastic search for maximum parsimony phylogenies
Source: PeerJ. 2013 Jun 25;1:e89. doi: 10.7717/peerj.89 (PMC3698465; doi:10.7717/peerj.89)
Supplement: Table S17 [file peerj-01-89-s017.pdf]

		Size of an input dataset						
		125	250	500	1,000	2,000	4,000	8,000
**Method**	NJ	0.1s	0.1s	0.1s	1s	8s	1m	8m 28s
	PAUP* (NNI)	1.1s	7.7s	54.4s	13m 59s	2h 13m	17h 42m	117h 24m
	PTree	6.4s	31s	2m 10s	6m 19s	22m 21s	2h 6m	10h 53m
	TNT (SPR)	0.7s	3s	14s	51s	5m 23s	57m 57s	4h 48m
	PAUP* (SPR)	14.4s	1m 50s	15m 14s	1h 12m	32h 5m	>1 month	–
	PAUP* (TBR)	32.3s	2m 28s	26m 1s	2h 32m	235h 41m	>1 month	–
