# Supplementary material for: PTree: pattern-based, stochastic search for maximum parsimony phylogenies
Source: PeerJ. 2013 Jun 25;1:e89. doi: 10.7717/peerj.89 (PMC3698465; doi:10.7717/peerj.89)
Supplement: Table S18 [file peerj-01-89-s018.pdf]

		Size of input dataset						
		125	250	500	1,000	2,000	4,000	8,000
**Method**	NJ	957	2,675	6,045	12,704	21,698	71,883	151,678
	PAUP* (NNI)	956	2,667	6,011	12,532	21,281	70,524	148,998
	PTree	934	2,614	5,907	12,328	20,976	70,301	148,515
	TNT (SPR)	934	2,592	5,850	12,214	20,743	69,545	146,906
	PAUP* (SPR)	939	2,602	5,870	12,226	20,736	69,508	–
	PAUP* (TBR)	937	2,591	5,850	12,183	20,693	69,380	–
