# Supplementary material for: PTree: pattern-based, stochastic search for maximum parsimony phylogenies
Source: PeerJ. 2013 Jun 25;1:e89. doi: 10.7717/peerj.89 (PMC3698465; doi:10.7717/peerj.89)
Supplement: Table S19 [file peerj-01-89-s019.pdf]

		Size of an input dataset						
		125	250	500	1,000	2,000	4,000	8,000
Method	NJ	0.1s	0.1s	0.1s	1s	8s	1m	8m 28s
	PAUP* (NNI)	2.1s	13.7s	2m 5s	19m 21s	4h 1m	38h 8m	166h 41m
	PTree	10s	41s	2m 17s	7m 59s	26m 32s	2h 12m	11h 21m
	TNT (SPR)	0.7s	3s	14s	51s	5m 23s	57m 57s	4h 48m
	PAUP* (SPR)	21.3s	3m 43s	31m 38s	2h 36m	78h	>1 month	–
	PAUP* (TBR)	51.3s	8m 45s	1h 2m	6h 44m	269h 44m	>1 month	–
