# Supplementary material for: PTree: pattern-based, stochastic search for maximum parsimony phylogenies
Source: PeerJ. 2013 Jun 25;1:e89. doi: 10.7717/peerj.89 (PMC3698465; doi:10.7717/peerj.89)
Supplement: Table S20 [file peerj-01-89-s020.pdf]

		Size of input dataset						
		125	250	500	1,000	2,000	4,000	8,000
**Method**	NJ	103.178	101.875	101.680	101.948	101.649	101.731	100.926
	PAUP* (NNI)	100.459	99.870	100.046	100.152	100.185	100.244	100.249
	PTree	100	100	100	100	100	100	100
	TNT (SPR)	99.574	99.265	99.313	99.223	99.080	99.051	98.991
	PAUP* (SPR)	99.656	99.222	99.332	99.281	99.129	99.155	–
	PAUP* (TBR)	99.607	99.170	99.220	99.187	99.056	99.100	–
